# Physical Activity and Snus: Is There a Link?

**DOI:** 10.3390/ijerph120707185

**Published:** 2015-06-25

**Authors:** Stéphane Henninger, Roland Fischer, Jacques Cornuz, Joseph Studer, Gerhard Gmel

**Affiliations:** 1 Department for Ambulatory Care and Community Medicine, University of Lausanne, Rue du Bugnon 44, 1011 Lausanne, Switzerland; E-Mails: Roland.Fischer@chuv.ch (R.F.); Jacques.Cornuz@chuv.ch (J.C.); 2 Alcohol Treatment Centre, Lausanne University Hospital CHUV, Rue du Bugnon 46, 1011 Lausanne, Switzerland; E-Mails: Joseph.Studer@chuv.ch (J.S.); Gerhard.Gmel@chuv.ch (G.G.); 3 Addiction Suisse, Av. Louis-Ruchonnet 14, 1003 Lausanne, Switzerland

**Keywords:** smokeless tobacco, youth tobacco use, education—youth prevention

## Abstract

The study aimed at assessing the link between physical activity (PA), sports activity and snus use among young men in Switzerland. Data from the Cohort Study on Substance Use Risk Factors (C-SURF) were used to measure PA with the International Physical Activity Questionnaire and sports activity with a single item. Multivariate logistic regression analysis was conducted to measure the association between snus use, PA and sports activity. Similar models were run for smoking and snuff use. Snus use increased in a dose-response association with PA (high level: OR = 1.72; 95% CI 1.16–2.55) and with individuals exercising once a week or more often (OR = 1.65; 95% CI 1.26–2.16; *p* < 0.001) or almost every day (OR = 2.27; 95% CI 1.72–3.01; *p* < 0.001) in separate models. Entered simultaneously, only sports and exercise maintained a basically unchanged significant dose-response relationship, whereas PA became non-significant. A non-significant dose-response relation was found for cigarette smoking and snuff use, indicating that the association with sport is specific to snus and not to tobacco use in general or smokeless tobacco in particular. This study showed that the association between snus use and sports is not specific to Nordic countries.

## 1. Introduction

Snus is a smokeless tobacco product that seems to gain in popularity with the Swiss population [1]. This Swedish-type smokeless tobacco is made of moist tobacco and food additives used as flavors. It is available either in a loose form or packed in portions and is placed between the upper lip and the gum. As soon as the nicotine is humidified by the saliva, it starts to diffuse trough the mucosa into the systemic circulation. Regular snus users are exposed to the addictive nature of that substance as much as regular cigarette smokers are, maybe more [2].

Different forms of smokeless tobacco exist in Switzerland. While the most popular form is nasal snuff, *i.e.*, mixed dry tobacco inhaled through the nose, chewing tobacco is quite uncommon. The prevalence of smokeless tobacco use was estimated by the Swiss tobacco control study in 2010 at about 2%–3% of the general population aged 14 to 65 years and up to 7.8% in men aged between 14 to 19 [3]. In comparison, the Swedish prevalence in men aged between 24–34 reached about 34% in 2002 [4]. Recently, based on the same sample as in the present study, *i.e.*, young men aged around 19–20 years in Switzerland, high prevalence rates of smokeless tobacco users were found with about 25% having used nasal snuff in the 12 months preceding the survey and 9% snus [5].

The sale of snus is currently prohibited in Switzerland and the entire European Union, except Sweden. The Swiss health agency justifies this legislation by arguing that this product is highly addictive and increases the risk of oral and pancreatic cancers [6]. A gateway phenomenon—snus users switching to cigarette smoking—is another argument based on scientific research conducted in Sweden and other Nordic countries where snus is commonly used [7]. Nevertheless, importing snus for personal use is allowed and it can be ordered online, without effective age control. The Swiss customs have observed a 56-fold increase in the quantity of oral tobacco imported from Sweden between 2004 and 2011, *i.e.*, from 484 to 27,410 metric tons [8].

After it was banned in the entire European Union in 1992 Sweden is the only country in EU that is authorized to manufacture and sell snus. This cultural exception was made when the country joined the Union in 1995. With about 17% of regular smokers, Sweden has the lowest smoking prevalence in the EU. This may be due to the many tobacco users who rather use snus than smoke tobacco. Furthermore, Sweden has the lowest lung cancer prevalence in the EU. Therefore, some experts in tobacco prevention promote snus as a safer alternative to cigarette. Considering that snus use is less hazardous than tobacco smoking, they argue that substituting snus with smoked tobacco may be beneficial to individual and public health [9,10,11]. In the same line of thought, the tobacco industry along with some physicians, have attempted to legalize the selling of snus in Switzerland, France and Germany [1,12]. Despite the fact that all such initiatives have failed so far, the pressure on the European Commission and governments is increasing.

Snus is very popular in sports, particularly in winter sports as ice hockey or downhill skiing [13,14,15,16,17]. In Switzerland, urine tests during the Ice Hockey World Championship performed by the Swiss anti-doping agency found that about 53% of the urine samples contained cotinine which is a metabolite of nicotine [18], suggesting a high prevalence of nicotine users among these ice hockey players. As smoking is harmful to lung capacities, which are particularly essential for professional athletes, the authors hypothesized that the majority of the nicotine was absorbed from using smokeless tobacco. Nicotine may enhance physical performances by rising blood pressure, heart rate, blood sugar and epinephrine secretion [19,20,21].

Physical activity (PA) measures all activities such as walking or carrying loads, be it during working hours or leisure time. Sport and exercise is different from physical activity because it is a specific part of everyday physical activity [15]. If you play ice hockey twice a week in addition to doing fitness training once a week with the aim of improving physical fitness and playing capacities, it is considered as a planned physical activity. In that regard, it is different from everyday physical activity. This means, for example, that a mason certainly spends more energy and has a higher physical activity (PA) level than a clerk playing football two hours a week.

First, the present study aims at testing whether there is an association between snus use and the level of physical activity, and sport and exercise in a population of young men in Switzerland, *i.e.*, in a non-Scandinavian country. Second, it also aims at testing whether the association with physical activity is mainly due to doing sports and exercise and not physical activity in general. Third, it investigates whether the association is found particularly for snus and not for smoking or the most common smokeless tobacco, namely snuff.

## 2. Methods

Army recruitment is mandatory in Switzerland for over 18-year-old males, who must undergo a two-hour assessment of their physical and mental aptitude for either military or civil service. During this medical test, virtually all young men were invited to participate in the Cohort Study on Substance Use Risk Factors (C-SURF) between August 2010 and November 2011 in the recruitment centers of Lausanne (French-speaking part of Switzerland), Windisch and Mels (both in the German-speaking part). While army recruitment centers were used to inform and enroll participants, the C-SURF study was independent from the army. During this period, 13,245 men were invited to participate in this study. Of these, 7564 (57.1%) men gave written informed consent. A few days after consenting, a one-hour long questionnaire was sent by email or by post to participants, and was filled out by 5987 men, thus reaching a 79.1% response rate. The research protocol (15/07) of the study was approved by the Ethics Committee for Clinical Research of the Lausanne University Medical School. The present study used data from the baseline assessment only.

To begin with, 244 participants (4.1% of the respondents) were excluded because of incomplete data on PA, sports activity or exercise, smokeless tobacco use or background variables. As daily snus users were few, snus use was categorized into “less than weekly” or “at least weekly”. Additionally, nasal snuff use was measured in order to test whether associations with physical activity and sport and exercise were unique to snus among smokeless tobacco users. Nasal snuff has been shown [5] to be the most common smokeless tobacco product used by this population. Snuff use was categorized into “less than weekly”, “weekly and more often”. As chewing tobacco is very uncommon in Switzerland [3], it was not included in the present study. Pictures in the questionnaire helped participants distinguish between the different forms of smokeless tobacco.

In order to measure self-reported PA, the short form of the International Physical Activity Questionnaire (IPAQ) was used. A multicenter study indicated that this instrument has good measurement properties, at least as good as other established self-reports [22]. Following the IPAQ guidelines for data processing and analysis [23], we removed 376 (6.3%) participants with unreliable data, e.g., subjects having more than 16 hours of physical activity per day. Reported PA of less than 10 min duration was assigned a zero value in 159 cases. As a result, the final analytic sample consisted of 5367 subjects.

Participants’ PA was categorized into three levels following the IPAQ guidelines: Low, moderate and high. The IPAQ guidelines suggest the metabolic equivalent or MET-minute unit to define these categories. Minutes of walking are calculated with a coefficient of 3.3, minutes of moderate PA (such as carrying light loads or playing golf) are multiplied by 4.0 and minutes of vigorous PA (as playing ice hockey or another intense sport practice) by 8.0. Daily physical activity and a sum of 3000 MET-minutes/week or vigorous PA at least three times a week achieving at least 1500 MET-minutes/week define the high level of physical activity. A moderate level of physical activity is described either as vigorous physical activity at least 20 min/day three days a week, or as moderate and walking activity during at least 30 min 5 days/week, or as any combination of physical activity amounting to at least 600 MET-minutes/day 5 days/week. Any activity below the limit of moderate PA was categorized into the low level. PA includes any activity during work, leisure time or at home. Although IPAQ is a widely used instrument, it does not distinguish between sports activity and other physical activities. Therefore, a single question served to assess sports activity or exercise: “Over the past twelve months, how often did you do sports or exercise?” Participants could choose between five answers: 1 = never, 2 = a few times per year, 3 = 1 to 3 times per month, 4 = at least once per week or 5 = almost every day. Sport and exercise were recoded into three levels: 1. Less than once a week; 2. Once a week or more; 3. Almost every day.

Based on the literature review, six different variables exploring the socio-demographic background of the participants and their smoking habits were used to adjust for potential confounders. Beside age and language (questionnaires were available in French and German), the socio-economic status was determined by the family income, which was divided into “below average”, “average” or “more than average”, according to the questionnaire of the European School Survey on Alcohol and Other Drugs [24]. Education level was assessed on the highest level achieved: “Primary schooling” (9 years), “vocational training” (>9–12 years) or “post-secondary” (13 years or more, including high school, which can be only 12 years in some Swiss cantons). Cigarette smokers were classified into “non-smokers”, “occasional smokers” and “daily smokers“. Finally, cannabis users in the past 12-months were categorized into “non-users”, “less than twice a week” users and “twice a week users or more often”.

Standard descriptive statistics were conducted to characterize the sample and to compare the levels of physical activity, sport and exercise according to snus and snuff use. A chi-squared test for categorical variables and t-tests for the continuous age variable were used.

Following to the descriptive analysis, unadjusted associations of physical activity and sport and exercise were tested using simple logistic regression models. Then, three multiple logistic regression models, adjusted for age, language, income, education level, cigarette and cannabis use, were performed. The first model compared physical activity levels according to snus use. The second model compared sport and exercise levels. Finally, the third model tested the associations of physical activity and sport and exercise with snus simultaneously, in order to examine the unique contribution of physical activity and sport and exercise. Low physical activity and less than weekly sport and exercise were set as reference groups. The same set of logistic regression models was performed for snuff use and cigarette smoking (occasional and daily smokers grouped together *vs.* non-smokers). This was done to investigate whether findings were specific to snus and not related to tobacco use in general or smokeless tobacco use in particular. For analyses on cigarette smoking, models were adjusted for the use of snus instead of smoking. Due to the small sample size, frequencies of snus and snuff use over the past 12 months were combined, and snus or snuff users over the past 12 months were compared to non-users. Odds ratios (OR) with a 95% confidence interval (95% CI) were presented. Analysis was conducted using the SPSS 21 software (IBM Corp., Armonk, NY, USA).

## 3. Results

Table 1 displays the demographic characteristics of the sample. Mean age (standard deviation) was 20.00 (1.24) at baseline. Of the 5367 analyzed questionnaires, 532 (9.9%) were categorized into the low level of PA, 1357 (25.3%) into the moderate and 3478 (64.8%) into the high level. In total, 372 (6.9%) used snus less than weekly and 112 (2.1%) weekly or more and 269 (72.3%) and 83 (74.1%) of them, respectively, were found in the high PA category. 

**Table 1:** Participants’ characteristics by snus use in the past 12 months.

	12-month Snus Use								
	Total (*N* = 5367)		No Use (*N* = 4883; 91.0%)		Less than Weekly Use (*N* = 372; 6.9%)		Weekly or More Use (*N* = 112; 2.1%)		*p*
	N	%	N	%	N	%	N	%	
Physical activity									0.001
Low	532	9.9	502	10.3	20	5.4	10	8.9	
Moderate	1357	25.3	1255	25.7	83	22.3	19	17.0	
High	3478	64.8	3126	64.0	269	72.3	83	74.1	
Sports and exercise									<0.001
Less than once a week	1515	28.2	1426	29.2	75	20.2	14	12.5	
Once a week or more	2226	41.5	2009	41.1	169	45.4	48	42.9	
Almost every day	1626	30.3	1448	29.7	128	34.4	50	44.6	
Age (Mean, SD)	20.00	1.24	20.02	1.25	19.75	1.19	19.68	0.87	<0.001
Language									<0.001
German	2381	44.4	2072	42.4	234	62.9	75	67.0	
French	2986	55.6	2811	57.6	138	37.1	37	33.0	
Family income									0.057
Below average	774	14.4	720	14.7	46	12.4	8	7.1	
Average	2203	41.0	2010	41.2	150	40.3	43	38.4	
Above average	2390	44.5	2153	44.1	176	47.3	61	54.5	
Highest achieved education									0.068
Primary schooling	2621	48.8	2361	48.4	193	51.9	67	59.8	
Vocational training	1556	29.0	1425	29.2	101	27.2	30	26.8	
Post-secondary	1190	22.2	1097	22.5	78	21.0	15	13.4	
Cigarette smoking									<0.001
Non-smoker	2834	52.8	2711	55.5	79	21.2	44	39.3	
Occasional smoker	1437	26.8	1213	24.8	174	46.8	50	44.6	
Daily smoker	1096	20.4	959	19.6	119	32.0	18	16.1	
Cannabis use									<0.001
Non-user	3720	69.3	3458	70.8	189	50.8	73	65.2	
Less than twice a week	1150	21.4	982	20.1	137	36.8	31	27.7	
Twice a week or more	497	9.3	443	9.1	46	12.4	8	7.1	

SD = Standard deviation.

**Table 2:** Participants’ characteristics by snuff use in the last 12 months (*N* = 5367).

	No Use (*N* = 4118; 76.7%)		Less Than Weekly Use (*N* = 1063; 19.8%)		Weekly or More Use (*N* = 186; 3.5%)		*p*
	*N*	%	*N*	%	*N*	%	
Physical activity							<0.001
Low	421	10.2	98	9.2	13	7.0	
Moderate	1081	26.3	248	23.3	28	15.1	
High	2616	63.5	717	67.5	145	78.0	
Sports and exercise							0.007
Less than once a week	1196	29.0	267	25.1	52	28.0	
Once a week or more	1653	40.1	492	46.3	81	43.5	
Almost every day	1269	30.8	304	28.6	53	28.5	
Age (Mean, SD)	20.08	1.29	19.74	1.02	19.64	1.05	<0.001
Language							<0.001
German	1678	40.7	579	54.5	124	66.7	
French	2440	59.3	484	45.5	62	33.3	
Family income							0.092
Below average	607	14.7	140	13.2	27	14.5	
Average	1705	41.4	413	38.9	85	45.7	
Above average	1806	43.9	510	48.0	74	39.8	
Highest achieved education							<0.001
Primary schooling	1943	47.2	562	52.9	116	62.4	
Vocational training	1194	29.0	305	28.7	57	30.6	
Post-secondary	981	23.8	196	18.4	13	7.0	
Cigarette smoking							<0.001
Non-smoker	2453	59.6	314	29.5	67	36.0	
Occasional smoker	880	21.4	477	44.9	80	43.0	
Daily smoker	785	19.1	272	25.6	39	21.0	
Cannabis use							<0.001
Non-user	3004	72.9	598	56.3	118	63.4	
Less than twice a week	726	17.6	365	34.3	59	31.7	
Twice a week or more	388	9.4	100	9.4	9	4.8	

SD = Standard deviation.

Concerning sport and exercise, 1515 (28.2%) exercised less than once a week, 2226 (41.5%) once a week or more and 1626 (30.3%) almost every day. In the latter category, 34.4% (*n* = 128) used snus less than weekly and 44.6% (*n* = 50) weekly and more. With regard to cigarette smoking, 79 (21.2%) of less than weekly snus users and 44 (39.3%) of weekly or more often snus users were non-smokers. Table 2 presents the descriptive characteristics of snuff users for comparison.

Results of the logistic regression models testing the associations of physical activity and sport and exercise with snus use are reported in Table 3. Models 1 and 2 tested the association of snus use with PA and sport and exercise, respectively. All were adjusted for age, language, income, education level, cigarette and cannabis use. The odds ratio (OR) for snus use increased in a dose-response association with physical activity, although only high PA significantly differed from low PA with an OR of 1.88 (95% CI 1.28–2.77) in the unadjusted model and an OR of 1.72 (95% CI 1.16–2.55) in model 1. Similarly, a dose-response association was found for sport and exercise with individuals exercising once a week or more (OR = 1.73; 95% CI 1.34–2.23; *p* < 0.001, for unadjusted model, OR = 1.65; 95% CI 1.26–2.16; *p* < 0.001, for model 2) and almost every day (OR = 1.97; 95% CI 1.51–2.57; *p* < 0.001, for unadjusted model, OR = 2.27; 95% CI 1.72–3.01; *p* < 0.001, for model 2) and being significantly more likely to use snus than those exercising less than once a week. In model 3, where PA and sport and exercise were entered simultaneously, the same dose-response association was found between snus use and sport and exercise. The OR remained significant and practically unchanged (OR = 1.59; 95% CI 1.21–2.08; *p* < 0.001, for once a week or more and OR = 2.10; 95% CI 1.57–2.81; *p* < 0.001, for almost every day sport or exercise). By contrast, the association between PA and snus use turned out to be non-significant and both moderate PA (OR = 1.19; 95% CI 0.77–1.83; *p* = 0.442) and high PA (OR = 1.36; 95% CI 0.91–2.04; *p* = 0.134) did not significantly differ from low PA.

With regard to analyses of cigarette smoking, the only significant association of PA was found for high PA as opposed to low PA (OR = 1.39; 95% CI 1.12–1.73; *p* = 0.003) in model 3. However, this difference was neither significant in the unadjusted model neither in model 1. By contrast, sport and exercise was significantly negatively related with cigarette smoking. Participants who reported doing sport and exercise once a week or more (unadjusted model: OR = 0.82; 95% CI 0.72–0.94; *p* = 0.003, model 2: OR = 0.85; 95% CI 0.73–0.99; *p* = 0.041, model 3: OR = 0.81; 95% CI 0.70–0.95; *p* = 0.008) and almost every day (unadjusted model: OR = 0.50; 95% CI 0.0.43–0.58; *p* < 0.001, model 2: OR = 0.53; 95% CI 0.45–0.63; *p* < 0.001, model 3: OR = 0.47; 95% CI 0.39–0.56; *p* = 0.008) were less likely to smoke cigarettes, as compared with those who reported doing sport and exercise less than once a week. 

**Table 3:** Logistic regression for snus, cigarette and snuff use in association with physical activity and sports and exercise.

	Unadjusted Model			Model 1			Model 2			Model 3		
	OR	95% CI	*p*	OR	95% CI	*p*	OR	95% CI	*p*	OR	95% CI	*p*
Snus use												
Physical activity			<0.001 ^a^			0.004 ^a^						0.227 ^a^
Low	1.00			1.00						1.00		
Moderate	1.36	0.89–2.07	0.151	1.29	0.84–1.99	0.239				1.19	0.77–1.83	0.442
High	1.88	1.28–2.77	0.001	1.72	1.16–2.55	0.007				1.36	0.91–2.04	0.134
Sports and exercise			<0.001 ^a^						<0.001 ^a^			<0.001 ^a^
Less than once a week	1.00						1.00			1.00		
Once a week or more	1.73	1.34–2.23	<0.001				1.65	1.26–2.16	<0.001	1.59	1.21–2.08	<0.001
Almost every day	1.97	1.51–2.57	<0.001				2.27	1.72–3.01	<0.001	2.10	1.57–2.82	<0.001
Cigarette smoking												
Physical activity			0.436 ^a^			0.638 ^a^						<0.001
Low	1.00			1.00						1.00		
Moderate	1.13	0.92–1.38	0.243	1.01	0.80–1.27	0.937				1.08	0.85–1.36	0.538
High	1.12	0.94–1.35	0.209	1.07	0.87–1.32	0.515				1.39	1.12–1.73	0.003
Sports and exercise			<0.001 ^a^						<0.001			<0.001
Less than once a week	1.00						1.00			1.00		
Once a week or more	0.82	0.72–0.94	0.003				0.85	0.73–0.99	0.041	0.81	0.70–0.95	0.008
Almost every day	0.50	0.43–0.58	<0.001				0.53	0.45–0.63	<0.001	0.47	0.39–0.56	<0.001
Snuff use												
Physical activity			0.002 ^a^			0.042 ^a^						0.041 ^a^
Low	1.00			1.00						1.00		
Moderate	0.97	0.76–1.24	0.799	0.92	0.71–1.19	0.521				0.90	0.69–1.17	0.417
High	1.25	1.00–1.56	0.050	1.13	0.89–1.42	0.322				1.11	0.87–1.42	0.389
Sports and exercise			0.001 ^a^						0.148 ^a^			0.144 ^a^
Less than once a week	1.00						1.00			1.00		
Once a week or more	1.30	1.11–1.52	0.001				1.18	0.99–1.39	0.053	1.16	0.98–1.37	0.093
Almost everyday	1.05	0.89–1.25	0.540				1.09	0.90–1.31	0.367	1.02	0.84–1.24	0.858

Model 1: Association of physical activity, adjusted for age, language, family income, highest achieved education, cigarette smoking (only for snus and snuff use), snus (only for cigarette smoking), cannabis use; Model 2: Association of sports and exercise, adjusted for age, language, family income, highest achieved education, cigarette smoking (only for snus and snuff use), snus (only for cigarette smoking), cannabis use; Model 3: Associations of physical activity and sports and exercise, adjusted for age, language, family income, highest achieved education, cigarette smoking (only for snus and snuff use), snus (only for cigarette smoking), cannabis use. OR = Odds Ratio; 95% CI = 95% confidence interval. ^a^ *p* value for overall factor.

Furthermore, no dose-response association of snuff use was found with PA and sport and exercise. In Model 1 high and moderate PA did not significantly differ from low PA. The same result was found when comparing those exercising once a week or more or almost every day with those exercising less than a week as shown in model 2. Model 3, testing the associations of PA and sport and exercise simultaneously, yielded similar non-significant results.

## 4. Discussion

The present study showed an association between snus use and a high level of physical activity and an even stronger association with sport and exercise in a population of young men in Switzerland. When tested simultaneously, odds ratios showed a significant association between snus and sport and exercise, but PA was no longer significant. This result suggests that the link between PA and snus use is mainly mediated through sport and exercise. Although sport and exercise is part of PA, it is important to distinguish the effects of sport from those of PA through other activities in order to better understand its association with substance use [15]. Indeed, the results of the present study showed that the association of snus use with sport and exercise is more relevant than with PA in general.

No association was found between nasal snuff and physical activity nor with sport and exercise, which indicates that the association does not exist with smokeless tobacco use in general, but with snus use in particular. This suggests that, despite the higher prevalence of snuff use, snus use may be associated with physical activity and sport or exercise. In addition, sport and exercise were negatively associated with cigarette smoking, indicating that the association between snus and sport and exercise was not due to tobacco use in general.

To our knowledge, this is the first study exploring the association between PA and snus use in Europe outside the Nordic countries. A previous study analyzed the relationship between sports activity and snus use in Finland [17]. The authors found odds ratios of 1.7 (95% CI 1.2–2.2) for light physical activity (less than 5 h/week), 3.6 (95% CI 2.8–4.8) for moderate physical activity (around 5 h/week) and 10.2 (95% CI 7.8–13.5) for regular competitive sports activity. In addition, the present study found a much higher proportion of participants categorized in the highest level of physical activity compared with the Finnish study. This result may be due to the use of a different measure of PA assessed by the IPAQ questionnaire, which allows a combination of moderate- and vigorous-intensity activities to meet the highest activity level [23]. Indeed, the association of snus use with PA could be identified already in descriptive statistics, *i.e.*, the percentage of individuals with the highest level of sports activity and exercise practice (almost daily practice): 30.3% in the total sample, compared with 29.7% with no snus use, 34.4% with less than weekly snus use and 44.6% with weekly snus use. Thus, the present study confirmed the dose-response relationship between physical activity and even more so with sport or exercising found by *Mattila et al.* [17].

The increasing amount of imported snus in Switzerland reported by the customs supports the hypothesis that snus gains in popularity in Switzerland and is used as in Nordic countries by the young and physically active population. Different studies conducted in France helped explain how this phenomenon of snus use was imported into other European countries from the Nordic countries. As snus is popular in winter sports, particularly among semi- and professional athletes [18], Bujon *et al.* [12,14] showed how this trend was brought into winter sports resorts where young people were in contact with these athletes. Young adults seeing their peers taking snus will be more likely to engage in the same behavior than if they had not been in contact with them. This could indicate that not only snus, but also its association with sports activities may be imported from Sweden. A previous Norwegian study found that the prevalence of snus use was almost three times higher for athletes competing in team sports such as ice hockey than individual sports (OR = 2.8, 95% CI 1.6 to 4.7) [25]. Like in Nordic countries, ice hockey is very popular in Switzerland and even if our study design does not permit us to identify specific sports activities, we speculate that the high popularity of winter sports such as ice hockey in Switzerland is a fertile ground for snus use.

Another outcome of our study is the high proportion of smokers that we found among snus users. Other studies found this association between smoked and smokeless tobacco products [26,27]. Although the aim of this study was not to explore the gateway phenomenon, results of the present study showed that only 21.2% of less than weekly snus users and 39.3% of weekly or more snus users were non-smokers compared with 55.5% among non-users of snus. Even though our study design does not permit a definite answer, this calls for caution as regards research supporting that snus could be a less harmful alternative to smoked tobacco. This seems unlikely to be the case in Switzerland, where snus is used in addition to smoked tobacco, and may not be used as a nicotine substitute for cigarettes or other smoked tobacco products.

One of the strengths of our study is the large sample size. Every Swiss man has to go through the mandatory army recruitment process, allowing us to obtain a representative sample of the whole 20-year-old male population in Switzerland. This study also has limitations. First, the cross-sectional design does not allow to draw causal conclusions. The longitudinal nature of the cohort study, however, will, in the near future, make it possible to measure the pathways from snus use to the use of other tobacco products, for example. Second, the present study is limited to a young male population and therefore no conclusions can be drawn about the behavior of females and older populations. Third, all data was obtained by means of a self-reported questionnaire. However, a previous study showed that young people tend to provide valid information about their use of tobacco products in self-reported questionnaires [28]. Fourth, no question about specific sports activities such as playing ice hockey was asked at baseline. Therefore no data was available to show whether snus use was specific to ice hockey players, for example. Nevertheless, self-reported data about sport and exercise enabled the assessment of regular sports activities in young men. Finally, the relatively high number of excluded questionnaires because of unreliable data following the IPAQ analyzing guidelines is another limitation of the study.

## 5. Conclusions

The present study confirmed that, as found in Nordic countries, snus use was associated with sport and exercise or with physical activity in Switzerland, where access to snus is legally prohibited. This may suggest that a particular marketing image associating snus with sports activities has been exported from Sweden to other countries. Therefore, snus use should be monitored, particularly among the physically active young population, and probably even more so among those practicing winter sports, in order to see whether it gains in popularity as it does in Nordic countries.

## References

[1] Swiss Parlement (2009). Produits de Substitution à la Cigarette. Avantages et Risques Présentés par le Tabac à Usage Oral. http://www.parlament.ch/f/suche/pages/geschaefte.aspx?gesch_id=20093786.

[2] Lee P.N. (2011). Summary of the epidemiological evidence relating snus to health. Regul. Toxicol. Pharmacol..

[3] Keller R., Radke T., Krebs H., Hornung R. Der Tabakkonsum der Schweizer Wohnbevölkerung in den Jahren 2001 bis 2010. http://www.tabakmonitoring.ch/Berichte/Tabakkonsum_Schweiz/Forschungsbericht/Gesamt_Tabakkonsum_10_dt.pdf.

[4] Stegmayr B., Eliasson M., Rodu B. (2005). The decline of smoking in northern Sweden. Scand. J. Public Health.

[5] Fischer R., Clair C., Studer J., Cornuz J., Gmel G. (2014). Prevalence and factors associated with use of smokeless tobacco in young Swiss men. Eur. J. Public Health.

[6] Meili B. (2008). Légalisation Éventuelle du Snus: Position de la CFPT.

[7] European Commission (2007). Health Effects of Smokeless Tobacco Products.

[8] Swiss Customs Importation of Snus from Sweden to Switzerland between 2004 and 2011. http://www.swiss-impex.admin.ch.

[9] Fagerström K., Schildt E. (2003). Should the European Union lift the ban on snus? Evidence from the Swedish experience. Addiction.

[10] Bates C., Fagerström K., Jarvis M.J., Kunze M., McNeill A., Ramström L. (2003). European Union policy on smokeless tobacco: A statement in favour of evidence based regulation for public health. Tob. Control.

[11] Molimard R. (2005). Le tabac sans fumée ou Snus, une réduction des risques liés au tabagisme. Courr. Addict..

[12] Bujon T. (2010). Le dopage et la réduction des risques: Le cas du snus dans le sport. Dépendances.

[13] Marclay F., Grata E., Perrenoud L., Saugy M. (2011). A one-year monitoring of nicotine use in sport: Frontier between potential performance enhancement and addiction issues. Forensic Sci. Int..

[14] Bujon T. (2008). Positifs à la nicotine. Psychotropes.

[15] Henchoz Y., Dupuis M., Deline S., Studer J., Baggio S., N’Goran A., Daeppen J.B., Gmel G. (2014). Associations of physical activity and sport and exercise with at-risk substance use in young men: A longitudinal study. Prev. Med..

[16] Connolly G.N., Orleans C.T., Blum A. (1992). Snuffing tobacco out of sport. Am. J. Public Health.

[17] Mattila V.M., Raisamo S., Pihlajamäki H., Mäntysaari M., Rimpelä A. (2012). Sports activity and the use of cigarettes and snus among young males in Finland in 1999–2010. BMC Public Health.

[18] Marclay F., Saugy M. (2010). Determination of nicotine and nicotine metabolites in urine by hydrophilic interaction chromatography-tandem mass spectrometry: Potential use of smokeless tobacco products by ice hockey players. J. Chromatogr. A.

[19] Benowitz N.L. (2009). Pharmacology of nicotine: Addiction, smoking-induced disease, and therapeutics. Annu. Rev. Pharmacol. Toxicol..

[20] Lagrue G., Cormier S., Lebargy F. (1996). La nicotine: Une substance psychoactive, un produit dopant?. Presse Med..

[21] Lagrue G. (2007). Fumeurs particuliers: Sportifs: Nicotine et tabac non fumé sont dopants: Tabagisme: Les sevrages difficiles. Concours Med..

[22] Craig C., Marshall A.L., Sjöström M., Bauman A.E., Booth M.L., Ainsworth B.E., Pratt M., Ekelund U., Yngve A., Sallis J.F. (2003). International physical activity questionnaire: 12-country reliability and validity. Med. Sci. Sport Exerc..

[23] (2005). Guidelines for Data Processing and Analysis of the International Physical Activity Questionnaire (IPAQ)—Short and Long Forms. http://www.ipaq.ki.se/scoring.pdf.

[24] (2011). Questionnaire on Substance Use. The European School Survey Project on Alcohol and Other Drugs. http://www.espad.org/uploads/documents/ESPAD_Questionnaire_2011.pdf.

[25] Martinsen M., Sundgot-Borgen J. (2014). Adolescent elite athletes’ cigarette smoking, use of snus, and alcohol. Scand. J. Med. Sci. Sports.

[26] Tomar S.L. (2003). Is use of smokeless tobacco a risk factor for cigarette smoking? The U.S. experience. Nicotine Tob. Res..

[27] Galanti M.R., Rosendahl I., Wickholm S. (2008). The development of tobacco use in adolescence among “snus starters” and “cigarette starters”: An analysis of the Swedish “BROMS” cohort. Nicotine Tob. Res..

[28] Post A., Gilljam H., Rosendahl I., Meurling L., Bremberg S., Galanti M.R. (2005). Validity of self reports in a cohort of Swedish adolescent smokers and smokeless tobacco (snus) users. Tob. Control.

